# Outcome and associated factors of high-risk human papillomavirus infection without cervical lesions

**DOI:** 10.1186/s12905-023-02764-8

**Published:** 2023-11-14

**Authors:** Ting Feng, Bei Cheng, Wenchao Sun, Yuhong Yang

**Affiliations:** 1 Department of Obstetrics and Gynecology, Hangzhou Women’s Hospital, No.369 Kunpeng Road, Shangcheng District, Hangzhou, 310008 Zhejiang Province China; 2 Department of Gynecologic Oncology, Women’s Hospital, School of Medicine, Zhejiang University, Hangzhou, 310008 Zhejiang Province China

**Keywords:** Human papillomavirus (HPV), Cervical intraepithelial neoplasia (CIN), Cervical cancer, Cancer-specific survival, Clearance, Viral load

## Abstract

**Objective:**

To study the outcome of human papillomavirus (HPV) infection in women with cervical pathology results of non-cervical intraepithelial neoplasia (CIN) or cervical cancer and positive high-risk HPV test, as well as analyze the associated risk factors affecting the outcome of infection.

**Methods:**

To investigate the outcome of high-risk (HR)-HPV infection in the female genital tract and analyze the associated risk factors affecting their outcome, a total of 196 women with positive HR-HPV test results and non-CIN or cervical cancer cervical pathology results were selected for follow-up at the Cervical Disease Clinic of the Obstetrics and Gynecology Hospital, Zhejiang University School of Medicine from January 2017 to March 2020. The follow-up interval was every 6 months, and both cervical cytology (TCT) and HR-HPV testing were performed at each follow-up visit. If the cervical cytology results were normal upon recheck and the HR-HPV test was negative, the woman was considered to be cleared of the HPV infection and was entered into the routine cervical screening population. When the repeat HR-HPV test remained positive after 6 months, the woman was defined as having a persistent HR-HPV infection. If HR-HPV persisted but the TCT results were normal, follow-up was continued. If HR-HPV persisted and the TCT results were abnormal, a colposcopy-guided biopsy was performed immediately. In this situation, if the histological results were still non-CIN or cervical cancer, the follow-up was continued. If the histological results confirmed the development of CIN or invasive cancer, then enter another study follow-up to further track its development and outcome, and the woman commenced the treatment process. The HPV infection clearance time was analyzed by the Kaplan-Meier method, and the comparison of the HPV clearance rate and infection clearance time between each of the different groups was performed using aχ^2^ test or Fisher’s exact test, as appropriate. After the univariate analysis, several significant factors were included in the Cox model and independent risk factors were analyzed.

**Results:**

A total of 163 women were enrolled in this study. The median age was 40.0 years (22–67 years) and the median follow-up time was 11.5 months (6–31 months). The spontaneous clearance rate of HR-HPV infection was 51.5%, and the median time to viral clearance was 14.5 months. Age and the initial viral load were high risk factors affecting the spontaneous clearance of HR-HPV infection. The factors significantly associated with HPV clearance rate and time to HPV clearance consisted of menopause and full-term delivery (*P* < 0.05).

**Conclusions:**

In women with normal or low-grade lesions on the cell smear, the spontaneous clearance rate of HR-HPV infection was 51.5% and the time to clearance was 14.5 months. Age and the initial viral load were independent associated factors affecting the spontaneous clearance of HR-HPV infection in the female genital tract. These findings suggest that non-young women or those with high viral loads have a higher rate of persistent HR-HPV infection. Thus, intensive screening should be recommended.

**Supplementary Information:**

The online version contains supplementary material available at 10.1186/s12905-023-02764-8.

## Introduction

It has been well established that cervical cancer is the fourth most common malignancy affecting women worldwide [1]. The International Agency for Research on Cancer estimates that there are more than 500,000 new cases of cervical cancer worldwide each year, with over 85% of these new cases and deaths occurring in developing countries [1].

In China, there are approximately 130,000 new cases of cervical cancer each year, ranking first among gynecological malignancies in China, with 20,000–30,000 women dying from cervical cancer each year. Among all malignant tumors, cervical cancer has the most definite factors associated with its development. In the mid-1990s, numerous epidemiological and molecular biology studies demonstrated that human papillomavirus (HPV) infection was required for the development of cervical cancer, and high-risk (HR)-HPVDNA could be detected in 99.7% of specimens collected from cervical cancer patients [2, 3]. Importantly, HR-HPV infection is a critically required pathogenic factor in the development of cervical cancer and its precancerous lesions [4, 5]. In terms of pathogenesis, cervical cancer is a sexually transmitted disease that can be prevented by blocking HPV transmission. Indeed, the World Health Organization (WHO) announced that cervical cancer is the only cancer that can be eradicated.

Papillomavirus is an envelope-free, double-stranded, circular DNA virus. HPV infection is very common in humans, with reported infections from birth in infants to elderly individuals over 80 years of age. During a woman’s lifetime, the chance of HPV infection in the genital tract is greater than 75% [6] and can be 40% or greater in young women in particular [7]. Most HPV infections are transient [8–11] and typically subside within a few months to 2 years post-infection. However, approximately 15% of HPV infections establish a persistent infection. In contrast, women with a persistent HPV high-risk infection have a 250-fold increased risk of developing a high degree of cervical epithelial lesions [12]. Proto-oncoprotein encoded by HR-HPVE6 and E7 genes is an important factor leading to cervical epithelial cancer [13, 14]. E6 protein can specifically bind P53 protein through E6-associated protein (E6-AP) to form a complex, which promotes rapid degradation of P53 protein and leads to uncontrolled cell cycle. Its effect is equivalent to P53 mutation, and E7 protein and retinoblastoma protein (pRB) have high affinity [13, 14]. The dissociation of E2F and pRB complex leads to immortalization and malignant transformation of cell cycle out of control [13, 14]. In addition, it has been well established that only persistent infections with HR-HPV strains can lead to the development of cervical cancer and its precursor lesions [4, 5]. However, differences in target populations, experimental design, and detection methods have resulted in different rates of natural clearance of HPV infection reported in the literature. The carcinogenic process of HPV is influenced by both host and environmental factors, and there are likely regional differences in the natural clearance of HPV infection. Thus, determining the pattern of natural progression of HPV infection in specific regions could help improve cervical cancer screening and vaccine effectiveness.

The risk factors associated with HPV infection have been widely reported. The more certain factors include sexual behaviors (e.g., number of sexual partners, age at first sexual encounter, and male sex), large number of births, HPV type, and age [15–17]. However, the correlation between factors such as smoking, contraceptive methods, HPV viral load and HPV infection remain controversial. The study by Molanoet al. followed 227 HPV-positive women with normal cervical cytology for a mean of 5 years [18], HPV-16 was found to have the lowest viral clearance compared to low-risk HPV types, whereas the viral clearance of HPV-31, − 33, − 35, − 52, and − 58 was between the two types. Moreover, viral load was not correlated with viral clearance. However, Dalstein et al. found that in HR-HPV positive women, viral clearance was higher in those with a low viral load (< 10 ng/L) compared with those with a high viral load (≥10 ng/L), suggesting that viral infection is likely to persist in those with a high viral load, thereby increasing the risk of lesions and lesion progression [12]. Furthermore, Song et al. found that the postoperative HPV persistence rate was significantly higher in patients with a high preoperative viral load than among those with a low preoperative viral load [19]. This suggests that viral load is an independent prognostic factor for HPV persistence and that patients with high viral load should be closely followed up after surgery. However, there are relatively few and poorly defined studies on the high-risk factors associated with HPV persistent infections.

This study aimed to investigate the outcome of HR-HPV infection in the female genital tract (e.g., clearance rate of HR-HPV, time to clearance, and rate of progression to non-cervical intraepithelial neoplasia (CIN)) by selecting women who tested positive for HR-HPV for the first time and who also had non-CIN cervical pathology and cervical cancer for follow-up. We also conducted a questionnaire survey of women with HR-HPV infection to obtain epidemiological information and analyze relevant risk factors affecting their outcome. These findings will help enhance our understanding of the natural outcome of HPV infection and its associated factors in Chinese women and provide a basis for proper clinical management of HPV-test positive women.

## Materials and methods

### Study subjects

All methods in our study were carried out in accordance with relevant guidelines and regulations or the declaration of Helsinki. The studies involving human participants were reviewed and approved by the Ethics Committee of Obstetrics and Gynecology Hospital of Zhejiang University School of Medicine. The patients/participants provided their written informed consent to participate in this study.

Those who attended the cervical disease clinic of the Obstetrics and Gynecology Hospital of Zhejiang University School of Medicine between January 2017 and March 2020 and met the following conditions were selected as study subjects: 1) positive results of a HR-HPV-DNA test (Hybrid Capture II, HC-II); 2) Thinprep Cytologic Test (TCT) results may be negative, or AUSCU or LISL, but whose the pathological results of colposcopy biopsy are non-CIN or cervical cancer; 3) not pregnant; 4) absence of a history of autoimmune diseases and oral immunosuppressive drugs; 5) no history of CIN or cervical cancer; 6) no history of cervical surgery; and 7) voluntarily participated in this study and signed an informed consent form. A total of 196 women met the inclusion criteria and were enrolled in the study.

### Collection for cervical exfoliative cell high-risk HPV-DNA test

No intravaginal medication or douching was permitted for 3 days before sampling, sampling was avoided during menstruation, no sexual intercourse was permitted for 24 hours before sampling, and an acetic acid/iodine solution was not to be applied before sampling. The tube was not shaken vigorously when opening the cap or breaking the small brush to avoid the spilling of reagents, which could affect the detection or cause the specimens to spill, causing contamination.

A second-generation hybrid capture test (Hybrid Capture II, HC-II) was used with a DIGENE HPV sampler (containing reagents that inhibit bacterial growth and maintain DNA integrity) to collect specimens from the cervical canal. The samples were sent to our central laboratory for HC-II testing. The wall of the test tube was labelled with the patient’s name or number, and the cap was tightened.

### Determination for cervical exfoliative cell HR-HPV-DNA test

The DNA from 13 oncogenic HPVs (i.e., HR-HPV types 16, 18, 31, 33, 35, 39, 45, 51, 52, 56, 58, 59, and 68), were detected. The test result of the viral load value was calculated as Relative Light Units/Standard Positive (RLU/PC) ratio. The Standard Positive value is 1.0 pg/mL. The viral load value ≥1 indicated a HPV positive result.

The initial viral load was divided into two groups: 1) a low load group, defined as a HR-HPV RLU/PC ratio of 1–99; and 2) a high load group, defined as a HR-HPV RLU/PC ratio was ≥100 [20].

### Collection for Thinprep cytologic test (TCT) assay

No intravaginal medication or douching was permitted for 3 days prior to sampling, sampling during menstruation was avoided, no sexual intercourse was allowed for 24 hours prior to sampling, and acetic acid/iodine could not be applied prior to sampling.

All cervical cell samples were collected using a special sampler for cervical cytology (TCT) and the sampler was placed in a vial with a cell preservation solution for rinsing.

The sample was then dispersed and filtered using a fully automated cytometer to reduce the amount of blood, mucus and inflammatory tissue residues for microscopic detection and diagnosis.

### Determination for TCT assay

The results of the TCT assay were determined in accordance with the Bethesda system (TBS) [21] as follows: 1) normal; 2) infection: protozoan, bacterial, fungal, viral, etc. infections; 3) reactive cell changes: in response to inflammation, injury, atrophy, hormones, etc.; 4) abnormal squamous epithelial cells: atypical squamous cells of undefined significance (ASCUS), atypical squamous cells not excluding high intraepithelial lesions, low grade squamous intraepithelial lesions (LSIL), high grade squamous intraepithelial lesions, suspected squamous carcinoma, definite squamous carcinoma; and 5) abnormal glandular epithelial cells: atypical glandular cells, atypical glandular cells tending to be neoplastic, suspicious adenocarcinoma, or adenocarcinoma.

### Colposcopy and cervical biopsy

The subjects were instructed to avoid intravaginal medication or douching, bimanual examination, or sexual intercourse for 72 h prior to the examination. For the examination, the cervicovaginal area was fully exposed with a vaginal speculum, and cervical secretions were gently wiped away with a cotton ball. Next, the focal length was adjusted, and the examined area was first observed roughly at a 10× low magnification microscope under white light. Next, a cotton ball soaked in 3% acetic acid was used to rub the cervicovaginal area to obtain a more precise observation. Finally, aniodine solution was applied, and a biopsy was collected from the negative iodine test area or suspicious lesions for pathological examination.

### Questionnaires

Each woman entering the study completed a questionnaire that included information regarding socio-demographic characteristics, lifestyle, menstrual and reproductive history, contraceptive practices, and aspects of sexual behavior. Medical staff conducted the survey through face-to-face or telephone questioning. During this process, the staff first explained the purpose, methods, and significance of this study to these respondents and obtained permission to conduct the survey. The completed questionnaires were collected on site and professional medical knowledge was provided to each of the subjects so that they could understand the causes of HPV infection, the characteristics of HPV as a virus, what was required for a formal follow-up, and the possible outcomes of a persistent HPV infection. At the same time, the patients were counseled to reduce their psychological stress and fear. A copy of the questionnaire form is attached for reference (Supplementary Table 1).

### Follow up

**Fig. 1:**
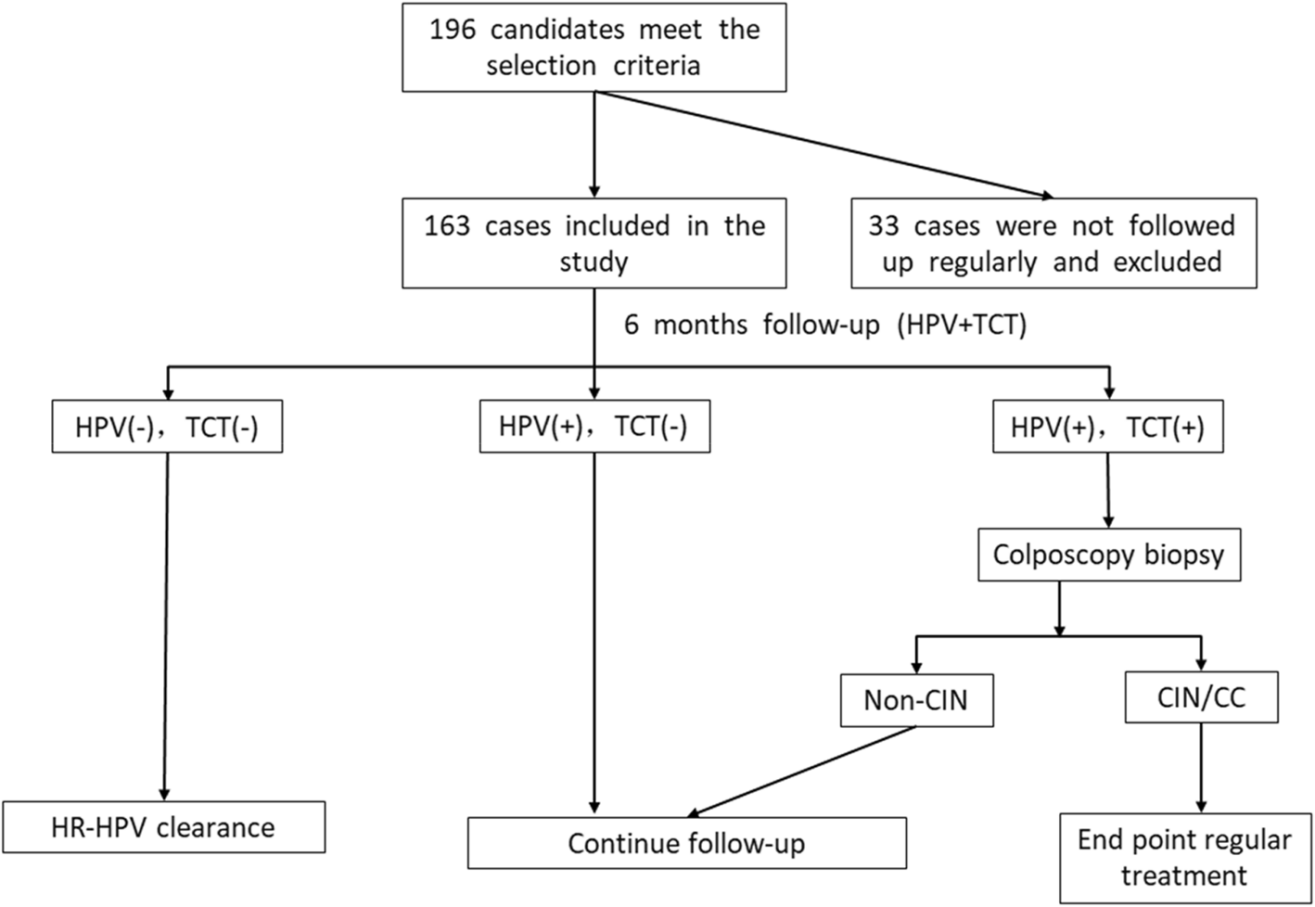
Flow chart of the follow-up procedure

Those who met the inclusion criteria between January 2017 and March 2020 were selected for this study. All data were collected between July 2017 and September 2020. During the follow-up, 33 of the 196 women who met the inclusion criteria were not followed up as required and thus excluded, resulting in a total of 163 women included in the study. No part of the cervix was treated with medication during the follow-up period. The follow-up interval was every 6 months, and both TCT and HR-HPV testing were performed at each follow-up visit. If the cervical cytology results were normal upon recheck and the HR-HPV test was negative, the woman was considered to be cleared of the HPV infection and was entered into the routine cervical screening population. When the repeat HR-HPV test remained positive after 6 months, the woman was defined as having a persistent HR-HPV infection. If HR-HPV persisted but the TCT results were normal, follow-up was continued. If HR-HPV persisted and the TCT results were abnormal, a colposcopy-guided biopsy was performed immediately. In this situation, if the histological results were still non-CIN or cervical cancer, the follow-up was continued. If the histological results confirmed the development of CIN or invasive cancer, the follow-up was terminated, then the woman enter another study follow-up to further track its development and commenced the treatment process. A flow chart depicting the follow-up process is shown in Fig. 1.

### Statistical analysis

All data were statistically processed using SPSS 13.0 statistical software. The HPV infection clearance time was analyzed by the Kaplan-Meier method, and the comparison of the HPV clearance rate and infection clearance time between each of the different groups was performed using aχ^2^ test or Fisher’s exact test, as appropriate. After the univariate analysis, several significant factors were included in the Cox model and independent risk factors were analyzed. A *P* value less than 0.05 was determined to be statistically significant.

## Results

### Subject demographic information

**Fig. 2:**
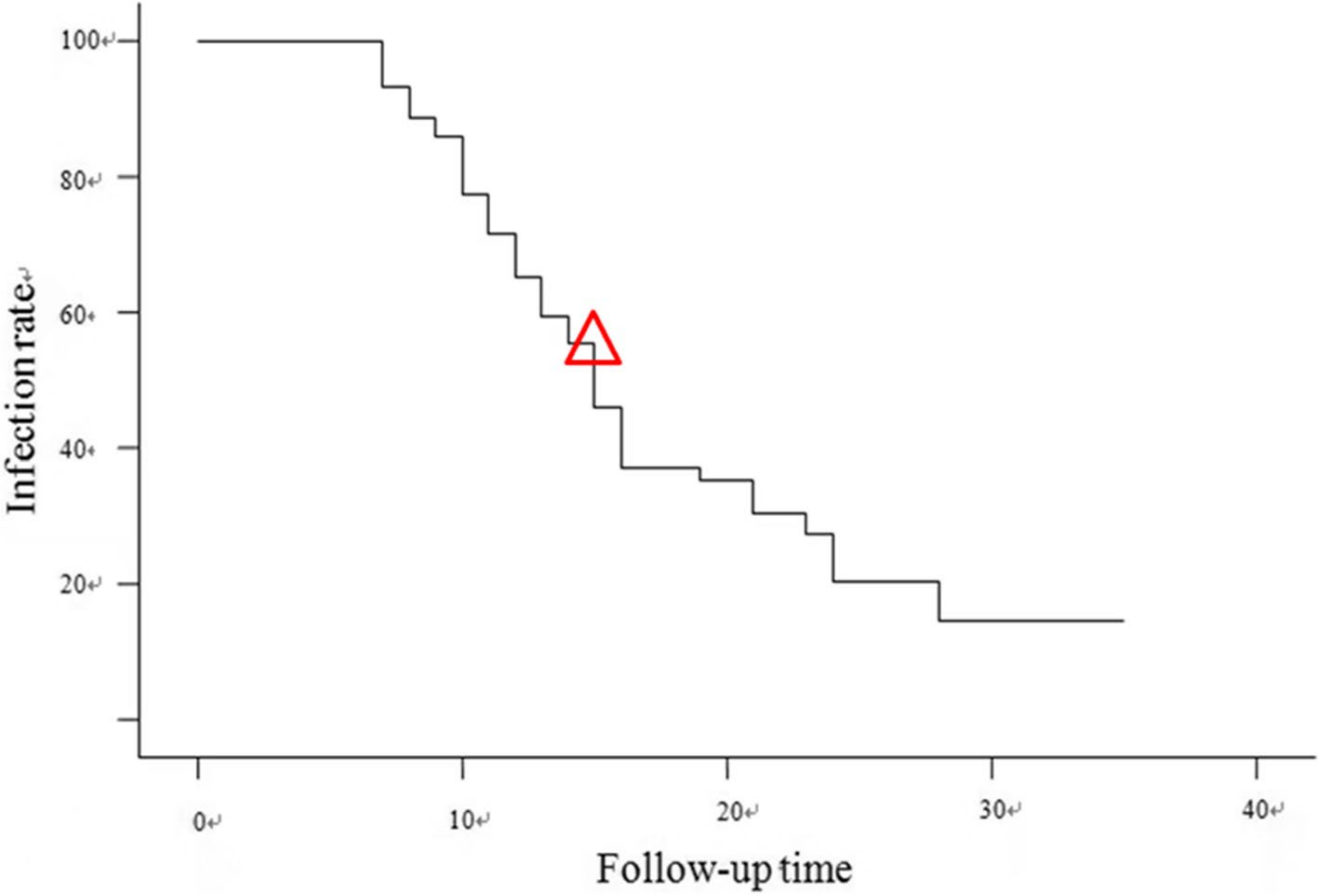
Relationship between the HPV infection rate and follow-up time. (△The median time of spontaneous clearance)

The median age of the 163 women enrolled in this study was 40.0 years (22-67 years, Fig. 2). The median follow-up time was 11.5 months (6-31 months, Fig. 2).

### Outcome of HR-HPV infection

The initial cell smears of 163 women showed that 85 cases were normal, whereas 78 cases were mildly abnormal (ASCUS or LSIL). By the end of the follow-up in this study, 84 of the 163 women tested negative for HR-HPV during follow-up, which represented a Spontaneous clearance rate of 51.5% for HR-HPV infection. The median time to the spontaneous clearance of HPV infection was 14.5 months (6–27 months). Of the 163 patients, 79 had persistent high-risk HPV infection and underwent colposcopy biopsy, of which 68 had no CIN or cervical cancer while 11 had CIN. There were 11 of 163 women who developed CIN (6.75%) and the median time was 13 months (6–31 months), including four with CIN I, four with CIN II, and three with CIN III.

### Relationship between age and spontaneous clearance of HPV infection

In the group younger than 35 years old, the spontaneous clearance rate of HPV infection was 63.2% (36/57), compared with 45.3% (48/106) in the group older than 35 years old. The difference between the two age groups was statistically significant (*P* < 0.001), as shown in Table 1.

**Table 1:** Correlation between age and HPV clearance rate (X^2^test)

Age	No. of cases (*n* = 163)	No. of HPV clearance cases	No. of HPV not clearance cases	X2	*P*
≤35 years	57	36	21	48.288	< 0.001
> 35 years	106	48	58		

**Fig. 3:**
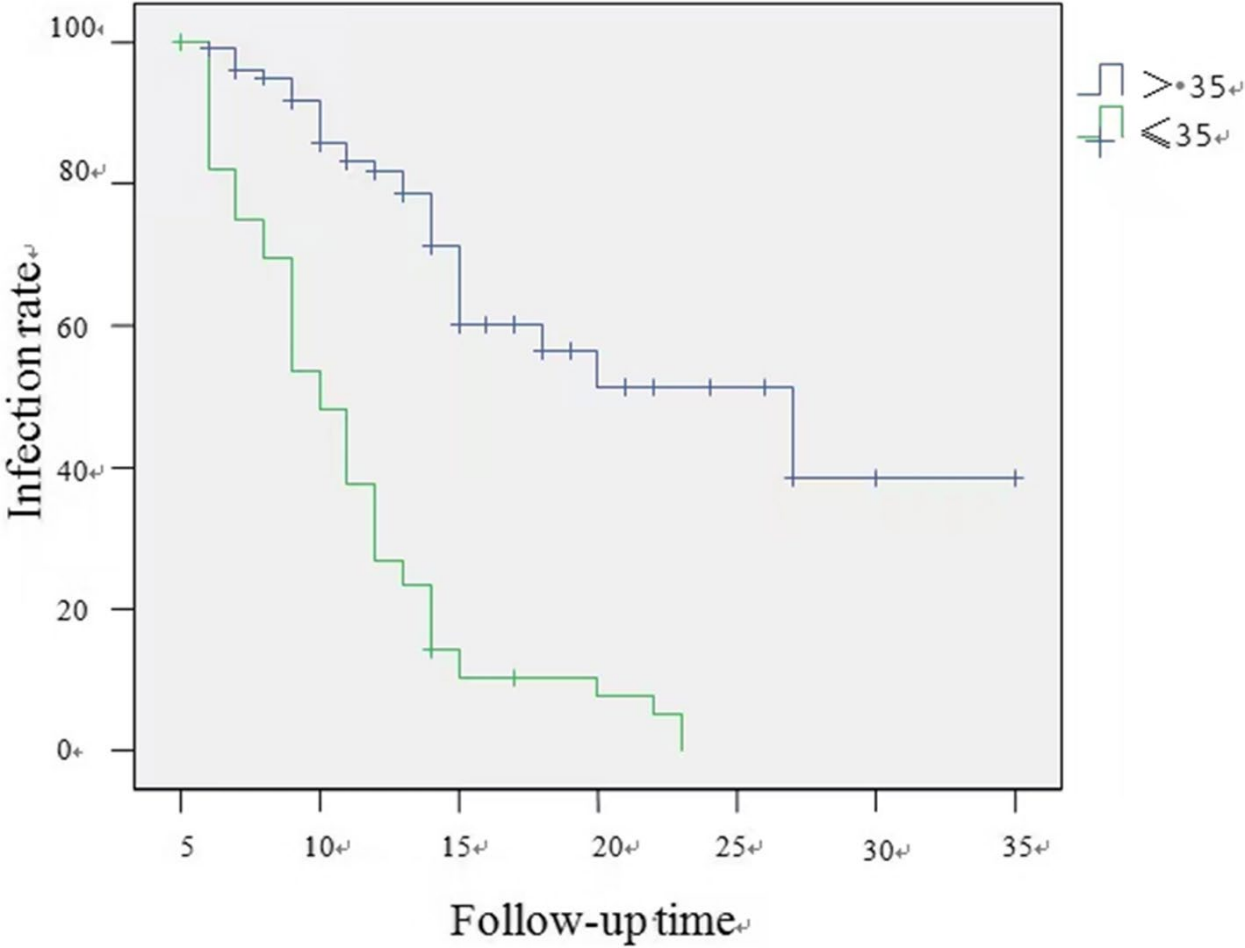
Correlation between age and HPV infection rate

In the group younger than 35 years old, the median time to the spontaneous clearance of HPV infection was 10.0 months (95% CI: 8.8-11.2 months). In contrast, in the group older than 35 years old, the median time to natural clearance of HPV infection was 27.0 months (95% CI: 14.6–39.4 months). The log rank test showed a statistically significant difference in the time to clearance of the HPV infection between the two age groups (*P* < 0.001), as shown in Fig. 3 and Table 2.

**Table 2:** Correlation between age and time to HPV clearance (log-rank test)

Age	No. of cases (*n* = 163)	Time of HPV clearance (Months)	*P*
≤35 years	57	10	< 0.01
>35 years	106	27	

### Relationship between initial cytological findings and viral load and the spontaneous clearance of HPV infection

**Fig. 4:**
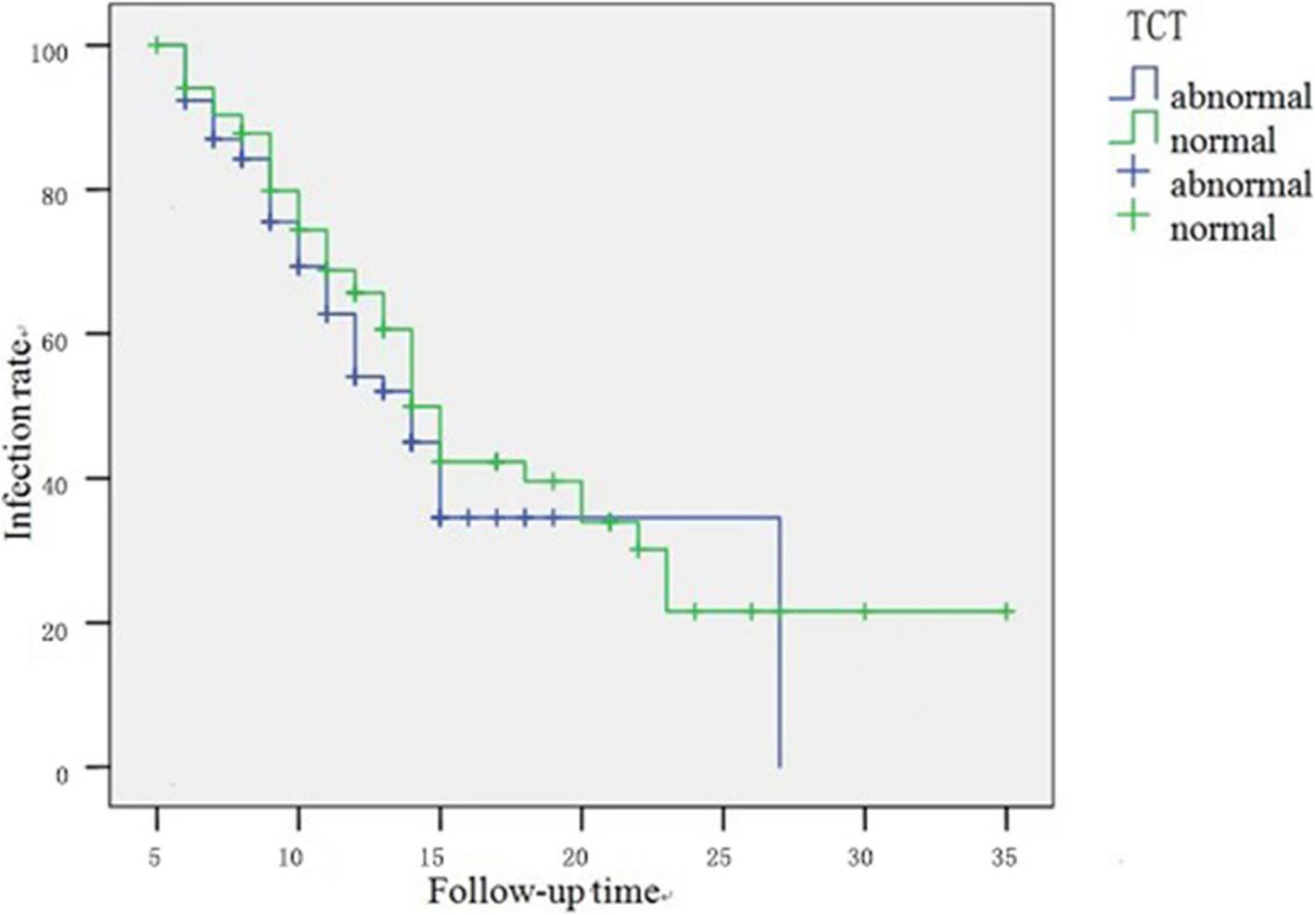
The relationship between TCT and HPV infection rate

**Fig. 5:**
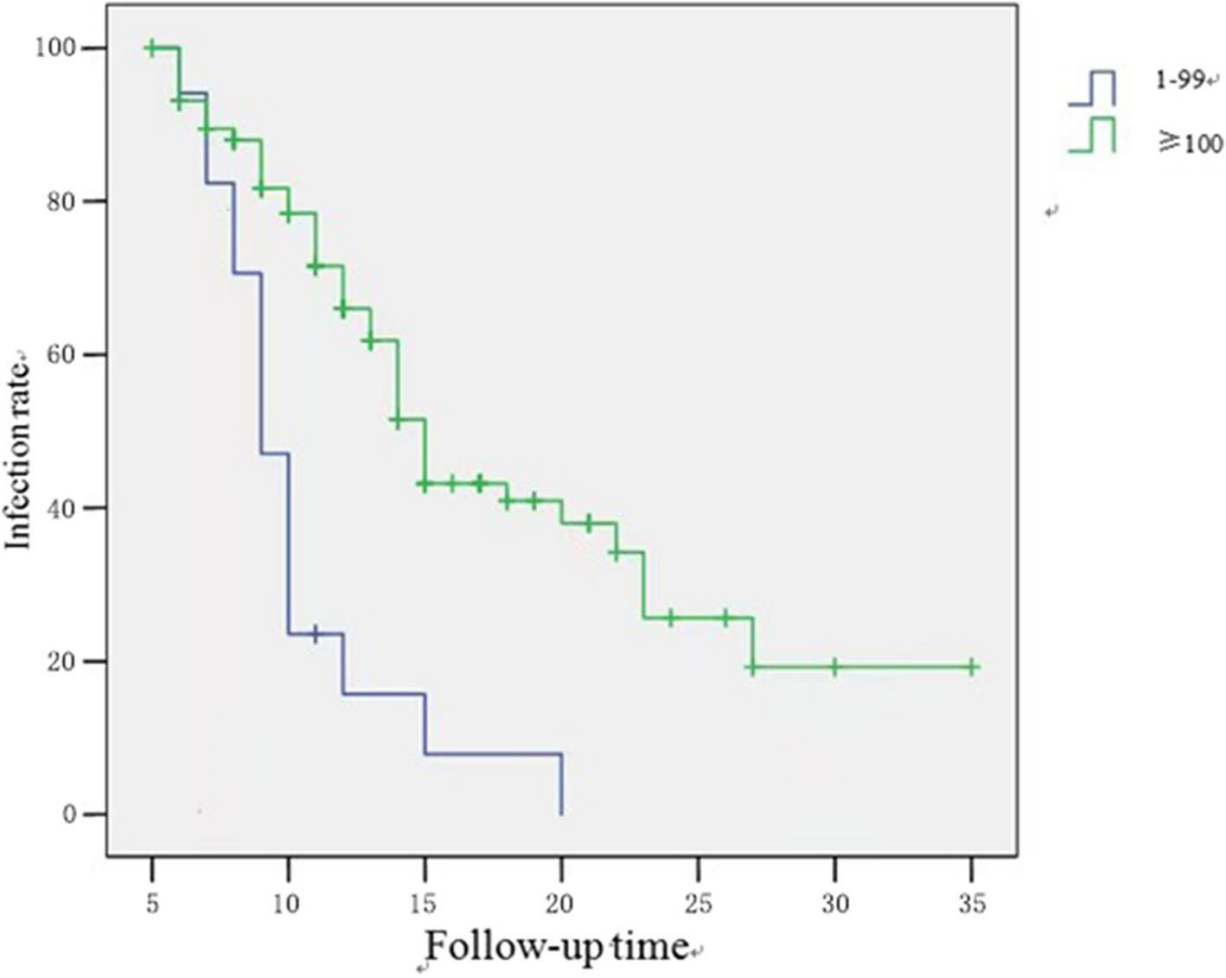
Relationship between virus load and HPV infection rate

The relationship between the initial cytologic findings, viral load and, and spontaneous clearance of HPV infection is shown in Table 3–4, as well as in Fig. 4–5. Both the χ^2^ test and Long-rank test revealed no statistically significant relationship between initial cytological outcome and HPV clearance rate and time to clearance. In contrast, there was a significant difference in the initial viral load between the low load group < 100 RLU/PC and the high load group ≥100 RLU/PC regarding the HPV clearance rate and clearance time (*P* < 0.001).

**Table 3:** Relationship between initial cytological findings and viral load and HPV clearance (X^2^ test)

Variables	No. of cases (*n* = 163)	No. of HPV clearance cases	No. of HPV not clearance cases	X2	*P*
Initial cytological findings					
Normal (NILM)	85	45	40	0.141	0.707
Anomalies (ASCUS/LSIL)	78	39	39		
Initial viral load (RLU/PC)					
1–99	74	50	24	13.78	<0.001
≥100	89	34	55

**Table 4:** Relationship between initial cytological findings and viral load and time to HPV clearance (log-rank test)

Variables	No. of cases (*n* = 163)	Time of HPV clearance (Months)	*P*
Initial cytological findings			
Normal (NILM)	85	14	0.33
Anomalies (ASCUS/LSIL)	78	14	
Initial viral load (RLU/PC)			
1–99	74	10	< 0.01
≥100	89	14

### Association of other factors with the natural clearance of HPV infection

Other factors associated with the HPV clearance rate and time to clearance included smoking or passive smoking, menopause, number of sexual partners, age at first sex, history of full-term delivery, and frequency of sexual intercourse. As listed in Table 5–6, the factors significantly associated with HPV clearance rate and time to HPV clearance consisted of menopause and full-term delivery (*P* < 0.05). The other factors were not significantly correlated with the natural clearance of HPV infection.

**Table 5:** Correlation of other factors with HPV clearance (X^2^ test)

Variables	No. of cases (*n* = 163)	No. of HPV clearance cases	No. of HPV not clearance cases	X2	*P*
Smoking or passive smoking					
Yes	64	31	33	0.404	0.525
No	99	53	46		
Menopause					
Yes	19	3	16	11.002	< 0.001
No	144	81	63		
Number of sexual partners					
1	110	58	52	0.193	0.66
≥2	53	26	27		
Age at first sex					
(years)	32	19	13	1.182	0.554
21–25	111	56	55		
≥26	20	9	11		
full-term delivery					
No	19	14	5	4.225	0.04
Yes	144	70	74		
Frequency of sexual intercourse (times/week)					
<2	103	52	51	0.123	0.726
≥2	60	32	28

**Table 6:** Correlation of other factors with HPV clearance ((log-rank test)

Variables	No. of cases (*n* = 163)	Time of HPV clearance (Months)	*P*
Smoking or passive smoking			
Yes	64	15	0.25
No	99	13	
Menopause			
Yes	19	27	<0.01
No	144	14	
Number of sexual partners			
1	110	14	0.82
≥2	53	14	
Age at first sex (years)			
≤20	32	14	0.22
21–25	111	14	
≥26	20	20	
Full-term delivery			
No	19	12	0.02
Yes	144	14	
Frequency of sexual intercourse (times/week)			
<2	103	15	0.96
≥2	60	14	

### Multifactorial analysis of factors affecting the spontaneous clearance of HPV infection

Age, initial viral load, menopause, and history of full-term delivery were included in the COX model in an ENTER manner that was significant in the univariate analysis. This regression equation was significant with χ^2^ = 65.596 and *P* < 0.01.

As seen in Table 7, age and initial viral load were found to be independent risk factors for the spontaneous clearance of HPV with regression coefficients of −0.071 and − 0.002, respectively. This finding indicates that both age and viral load were negatively associated with the risk of clearance (i.e., the older the age, the less likely it was that the virus could be cleared. Moreover, the higher the initial viral load, the lower the likelihood of viral clearance.

**Table 7:** Multifactorial analysis of factors affecting the natural clearance of HPV infection

	B	SE	Wald	df	Sig	Exp(B)	95.0% CI for Exp (B)	
							Lower	Upper
Age	**−.071**	.019	13.841	1	**.000**	.932	.898	.967
Viral load	**−.002**	.001	9.158	1	**.002**	.998	.996	.999
Full-term birth	−.351	.321	1.194	1	.275	.704	.375	1.321
Menopause	−.364	.679	.289	1	.591	.695	.184	2.626

## Discussion

### Spontaneous clearance of HPV infection

Both epidemiological data and clinical studies have demonstrated that most HPV infections, as well as the corresponding precancerous cervical lesions (i.e., low-grade lesions), resolve spontaneously. However, the time to spontaneous clearance of HPV infection varies from 7 to 18 months due to different populations included in each study, methods of HPV testing, and modes of follow-up. Dalstein et al. conducted a prospective study of 781 women with normal/ASCUS/LSIL cervical cytology in 2003 [22].These 781 women were tested for HR-HPV using a second-generation hybrid capture assay (HC-II) with follow-up every 6 months for a mean follow-up of 22 months. The results revealed a mean duration of infection of 7.5 months (3 to 42 months) in HR-I HPV-positive patients, with > 50% of patients clearing the infection within 7.5 months. In 2009 Jaeman Baeetal. reported that 224 high-risk HPV positive Korean women with normal cervical cytology were reported. After an average follow-up of 20 months, the natural clearance rate of the virus was 68.3%, and the median clearance time was 7.5 months [23]. Moreover, in 2007, Bulkmans et al. reported that 865 high-risk HPV-positive women with normal cervical cytology had a spontaneous clearance rate of 65% and a mean clearance time of 18 months after a mean follow-up of 40 months [10].However, there are few studies on the outcome of HR-HPV infection in Chinese populations. In this study of 163 women with normal or mildly abnormal HR-HPV smears, the spontaneous clearance rate of HR-HPV infection was 51.5% and the time to clearance was 14.5 months when followed up for a mean of 11.5 months. The lower clearance rate of HR-HPV in the study compared with that of previous reports may be explained by the following analysis: 1) there were differences in the HPV clearance rates among the different study populations; 2) there was a short follow-up time (average of 11.5 months, with the shortest follow-up time of only 6 months), which inevitably affected the clearance rate; 3) the sample size was small. We will continue to follow up these patients and collect data regarding their HPV outcome after 1.5 to 2 years. We also intend to keep increasing the sample size of the studied patients to further understand the spontaneous clearance of HPV among Chinese women.

### Factors associated with the spontaneous clearance of HR-HPV infection

It has been suggested that a high-risk sexual lifestyle, including young age of sexual initiation, multiple sexual partners, male sexual activity, high number of births, and frequent sexual intercourse, as well as a history of smoking or passive smoking, contraceptive method, menopause, history of childbirth, and initial cytological findings, constitute the main risk factors affecting the spontaneous clearance of HR-HPV infection in women [24–26]. However, various studies provide differing views on the potential roles of these factors. The study by Maucort-Boulch et al. reported a significant difference in persistent high-risk HPV infection caused by smoking or passive smoking more than 20 cigarettes/day and less than or equal to 10 cigarettes/day between these two groups [23]. In addition, reproductive factors may also be associated with HPV infection. Vaccarella et al. suggested that the risk of HPV positivity was greater among Nulliparous than in menstruating women [27].The present study did not find any association between the number of sexual partners, frequency of sexual intercourse, age at first sex, and the spontaneous clearance of HPV infection, which may be related to the relatively conservative nature of Chinese women, who may have reservations about participating in an investigation of sexual behavior and did not provide accurate information. In the multifactorial analysis of this study, menopause, full-term delivery showed correlation with the clearance of HPV infection, nevertheless, smoking or passive smoking, and initial cytologic findings did not show any correlation with the clearance of HPV infection. This finding may be related to the fact that the population in this study was from the southern region of China, where there are fewer women who are smokers. In addition, there have recently been increased efforts to ban smoking in public places, and smoking or passive smoking was not shown to be associated with HPV clearance in this study. Furthermore, in our initial analysis of cytological outcomes, the results were similar between the cytologically normal population and the ASCUS and LSIL populations, with no significant differences. Thus, we suggest that in the asymptomatic female population, the cytological abnormalities are mainly minor lesions, which may not have a significant impact on the HPV clearance curve.

Among the many factors that affect the natural clearance of HR-HPV infection in previous studies, age is another factor that has been extensively studied. Several studies have found that while age represents the most important factor affecting the distribution of HPV infection rates [7], it also influences the spontaneous clearance of HR-HPV infection. Rousseau et al. followed the outcome of HPV infection in 252 patients with a pathological diagnosis of CINI with cervical conization and found that age was the primary predictor of HPV clearance [28]. Maucort-Boulchet al. also reported a high rate of persistent infection in older women infected with HPV in a cross-sectional study [23]. In the present study, a significant difference was observed in the clearance rate and time to clearance of high-risk HPV between women aged less than 35 years and those aged greater than 35 years, a phenomenon that may be related to the decreased ability to clear recent infections or suppress latent HPV due to age-related declines in immune function [29]. Thus, the clinical significance of high-risk HPV positivity in screening increases with age. Thus, immediate cytological screening should be recommended for middle-aged and elderly women with high-risk HPV positivity due to their greater chance of persistent infection. If the cytology is negative, HPV testing and cytology can be repeated in 6 months. If repeat HPV testing reveals a persistent HR-HPV infection, immediate colposcopy should be recommended, even if the cytology results are negative.

Viral load is another high-risk factor for possible persistent HPV infection. Dalstein et al. studied 781 women with normal and different grades of cervical lesions, who were followed-up every 6 months with a median follow-up of 22 months [12]. The authors found that the viral clearance rate was higher among those with a low viral load (< 10 ng/L) compared to those with a high viral load (≥10 ng/L). This suggests that viral infection is likely to persist in those with a high viral load, thereby increasing the risk of lesions and lesion progression. Song et al. studied 67 patients with high-risk HPV-positive CIN II/III, in which all cases were treated with cervical conization, and postoperative pathology confirmed no lesions at the incision margins [19].The HPV viral load (HC-II method) was measured before and 6 months after surgery and revealed that 43.8% of patients with a preoperative viral load > 500 RLU/PC had persistent HPV infection after surgery, whereas only 9.8% of patients with a viral load < 500 RLU/PC had persistent HPV infection after surgery. This finding indicates that viral load is an independent prognostic factor for persistent HPV infection, and patients with a high viral load should be closely followed up after surgery. However, there are also some scholars with the opposite view Onan et al. studied 94 patients with cervical lesions, including 47 cases of CINI, 27 cases of CINII, and 20 cases of CINIII, and found that the positive rate of HPV infection increased with the cervical lesion grade, but there was no corresponding trend of increasing viral load [30]. Thus, the authors concluded that there was no correlation between viral load and cervical lesion grade. In the present study, the initial viral load was divided into two groups: 1) a low viral load group with RLU/C < 100.0 and 2) a high load group with RLU/C ≥ 100.0.Significant differences were found in the HR-HPV clearance rate and clearance time between these two groups, and the multifactorial analysis confirmed that the initial viral load was an independent correlate of the spontaneous clearance of an HPV infection. In addition, 11 of 163 women followed up in this study developed CIN, all of whom were in the high viral load group. Therefore, the initial viral load may be clinically considered for screening women with high-risk factors for the development of CIN and as a follow-up indicator for women with high-risk HPV infection and normal cytology. However, these findings remain to be demonstrated with large samples to make reliable conclusions. At the same time, this study used the HC-II method to detect high-risk HPV, and the viral load was among the obtained results; however, the specific HPV type could not be identified as precisely as the PCR method, making it impossible to determine whether the persistent HR-HPV infection observed in this study was the persistence of the same HPV type or the emergence of another type of HPV infection. Therefore, follow-up studies on HPV viral load should apply novel viral quantification methods capable of typing (e.g., dynamic PCR methods) and establish standard specimen collection and testing methods to facilitate more effective statistical analyses.

## Conclusion

Among women with normal cell smears or low-grade lesions, the spontaneous clearance rate of HR-HPV infection was 51.5% following a mean follow-up of 11.5 months, with a clearance time of 14.5 months. Age and initial viral load are independent correlates of the spontaneous clearance of HR-HPV infection in the female genital tract, suggesting that HR-HPV infection in non-young women or those with a high viral load is associated with a higher rate of persistent infection. Therefore, an aggressive screening approach is an appropriate recommendation. Menopause and full-term delivery were significantly correlated with clearance of HPV infection.

### Supplementary Information

**Additional file 1.** HPV Risk Factors Questionnaire

## Data Availability

The datasets used and analyzed during the current study are available from the corresponding author on reasonable request.

## Abbreviations

HPV: Human papillomavirus
CIN: Non-cervical intraepithelial neoplasia
E6-AP: E6-associated protein
HC-II: Hybrid Capture II
TCT: Thinprep Cytologic Test
ASCUS: Atypical squamous cells of undefined significance
LSIL: Low grade squamous intraepithelial lesions
