# Supplementary material for: Outcome and associated factors of high-risk human papillomavirus infection without cervical lesions
Source: BMC Womens Health. 2023 Nov 14;23:599. doi: 10.1186/s12905-023-02764-8 (PMC10644444; doi:10.1186/s12905-023-02764-8)
Supplement: Supplementary file 1 — Additional file 1. HPV Risk Factors Questionnaire [file 12905_2023_2764_MOESM1_ESM.docx]

# HPV Risk Factors Questionnaire

## Name Contact Number Patient No

**(A) Lifestyle**

**A1** Age

**A2** Marital Status

Married (1) Divorce (3) Spinsterhood (5)

Widowed (2) Separation (4) Cohabit (6)

**A3** occupation

**A4** Educational background (subject to diploma)

**A5 a.** Are you a smoker or a former smoker? (Definition of smoking: "≥1 cigarette/day for more than one year")

No 0 Yes 1

If the answer is "0", go to A6

**a.** How many years have you smoked in total? (year)

b. How many cigarettes do you smoke per day on average? (branch)

#### A6 a. Are you a passive smoker in your daily work or life or have you ever been a passive smoker? (Passive smoking, commonly known as second-hand smoke, refers to non-smokers who breathe the smoke exhaled by smokers for more than 15 minutes per day for more than 1 day in a week)

No 0 Yes 1

If the answer is "0", go to A7

1. How many days per week do you be exposed to passive smoking? (Day)
2. How much time do you suffer passive smoking per day on average? (minutes)

How many passive cigarettes do you be exposed to per day on average? (branch)

1. How long have you been exposed to passive smoking？（year）

**A7** Per capita monthly household income (Yuan)

< 201 0 201～500 1 501～1000 2

1001～2000 3 2000～3001 4 3001～5000 5

≥5000 6

**(B)** [childbearing history](javascript:;)

**B1.** Marital/reproductive history

**a.** Marriage age (if not already married, please fill in "0")

**b.** reproductive history

**c.** Age of first pregnancy

**B2.** The main form of contraception used in the last two years

**B3.** History of Oral Contraceptives

1. Have you ever take Oral [contraceptive](javascript:;) No 0 Yes 1

If the answer is "0", go to C1

a . Oral Contraceptives Category

Short-acting Oral [contraceptive](javascript:;) (e.g. Marvalon,etc.) 1

Long-acting Oral [contraceptive](javascript:;)（e.g.Left-hand long-acting film,etc.） 2

Visiting Oral [contraceptive](javascript:;) (Or Emergency contraceptive pills, rapid-active [contraceptive](javascript:;) pills, e.g. Number 53 Visiting Oral [contraceptive](javascript:;), Yu Ting, etc) 3

1. Frequency of Oral [contraceptive](javascript:;) (times/month)[Example: once a year, can be expressed as 1/6 (times/month)] (For occasional use, please fill in "0")
2. Total number of years of oral contraceptives (years) (for occasional use, fill in "0")

**（C） Diseases History**

C1. History

1. Have you ever suffered from autoimmune diseases such as systemic lupus erythematosus, rheumatoid arthritis, hyperthyroidism, etc

No 0 Yes 1

1. Have you ever suffered from benign neoplastic diseases (such as uterine fibroids, breast fibromas, hemangiomas, schwannomas, etc.)

No 0 Yes 1

1. Have you ever suffered from malignant neoplastic diseases

No 0 Yes 1

1. Have you ever had a hysterectomy?

No 0 Yes 1

1. Have you ever taken estrogen (tablet, injection, patch) for menopause or other reasons? Years of menopause
2. No 0 Yes 1

If the answer is "0", go to i

1. If yes, how old were you when you first started using estrogen? (age)
2. How many years did you use estrogen? (year)
3. Are you taking progesterone with estrogen due to menopause or other reasons?

No 0 Sometimes 1 [at the same time](javascript:;) 2

1. Have you had a pap smear?

No 0 Yes 1

If the answer is "0", go to C2

1. How many years ago did you have your last cervical scrape(Year)

**（D）Sexual history**

**D1.** How old were you when you started having sex?

**D2.** How often do you have sex

<1 times/month 0 1-2 times/month 1 1 times/week 2 2-3 times/week 3 >3 times/week 4

**D3.** How many sexual partners have you had so far?

**D4.** If you have only had one sexual partner for how many years? (year)

**D5.** Do you use other methods besides genital intercourse?

No 0 Yes 1

If the answer is "0", go to D7

**D6.** If there were other ways of having sex ,it’s： Mouth and genitalia 1 Anus - genitalia 2

others 3

Date:

Investigator:

[**Questionary**](javascript:;)

| **Project**  **Time** | | **HPV Result** | | **TCT** | | **Pathology** | |
| --- | --- | --- | --- | --- | --- | --- | --- |
|  |  | **Time** | **Result** | **Time** | **Result** | **Time** | **Result** |
| **First visit** | |  |  |  |  |  |  |
| **Subsequent visit** | **1** |  |  |  |  |  |  |
|  | **2** |  |  |  |  |  |  |
|  | **3** |  |  |  |  |  |  |
|  | **4** |  |  |  |  |  |  |
|  | **5** |  |  |  |  |  |  |
